# Selections and Abstracts

**Published:** 1903-11

**Authors:** 


### SELECTIONS AND ABSTRACTS.

### What is Necessary to the Early Treatment of Insanity? *

The greatest difficulty in the way of progress in the medical treatment of the insane is that we are prone to think of the future in terms of the present. When we consider the doing of an act in the future, we always have a picture of the present in our minds. Therefore, when we try to consider insanity as a manifestation of disease, just as we would scarlet fever or pneumonia, there comes up in our minds the picture of the wild-eyed individual, with matted hair and torn garments, whose presence fills us with superstitious fear; and we instinctively think of him as the victim of demoniac possession; forgetting that, like the scarred victim of smallpox, or the loathsome subject of tertiary syphilis, as we used to see him, he is simply the result of the conditions our ignorance and superstition have placed him in. Unfortunately, too, we are further hampered by the fact that present conditions are in a great measure beyond our control, because they have been molded into public opinion and fixed by statute. Public opinion does not seem to realize that if we treated the insane man as we do any other sick man, he would cease to exist as we now imagine him, an unsocial being. It is true that there will always be a class of individuals who are essentially insane from the time of their birth, and who, as they grow older, become unsocial beings like Guiteau and Prendergast. But such people are the exceptions, and they ought not to be the basis for the consideration of what should be the proper treatment of the acutely insane any more than should the chronic, quiet and well-behaved dement, who is, at present, the main object of sentimental and philanthropic regard. My experience has taught me that while theoretically and sentimentally the insane are regarded as sick people, practically they are not, as is

*Read by H. A. Tomlinson, M.D., Superintendent of St. Peter State Hospital, St. Peter, Minn., before the Aberdeen District Medical Society, Aberdeen, S. D., Sep. 15, 1903.

shown by the persistence in most States of laws which gives the insane a quasi-criminal status. Besides, insanity is not known to the public except in its most extreme manifestations, and the patient is not placed under treatment because he is insane, but because his conduct has made him dangerous to the community. Consequently these unfortunate people have been looked upon by the world very much as was the leper by the Jew. They have been thought of as beyond the pale of human sympathy and interest, as objects of horror and dread; to be pitied in an indefinite sort of way, but always at a distance. We have constantly to struggle against the inertia of custom and the influence of tradition and superstition in keeping alive medieval ideas concerning what is constituted in insanity. These difficulties are well illustrated in the attitude of the public mind, as well as that of physicians who have not become familiar with the insane by association. The fact of insanity is admitted without appreciation of the significance of the mental perversion, or any realization that the relation of the insane man to his fellows is changed by his condition; therefore, those by whom he is surrounded are disposed to accept his statement of his relations, and to be influenced by what he says. Again, in considering the necessities for his care and treatment, nothing is appreciated but his clothing, housing and feeding. The acutely insane man is thought of as a sort of wild animal to be kept out of sight; while the chronic, quiet and well-behaved patient, for whom nothing need or can be done, receives the sympathy and interest of the sentimental and philanthropic, because there is nothing shocking to the sensibilities in his behavior, or offensive in his appearance; besides, all of his needs are supposedly familiar to the general public.

In order to understand the weight of these influences, as they interfere with progress, it will be necessary to review briefly the history of insanity and the care of the insane, as it developed in this country out of the traditions brought from the old world. Until within a comparatively recent period the mind was conceived of as an entity, independent of the body and its functions. Therefore, the condition insanity was irremediable, without hope for improvement, except through the intervention of divine providence. It is true also, that this belief was fostered and kept alive by religious belief and the teachings of metaphysics. Among savages, the insane man was either looked upon with reverence as under the special protection of deity; or, if disturbed and violent, as possessed of an evil spirit, and disposed of accordingly. His treatment among civilized people did not differ much in kind, until, in the middle ages, he was turned over to the care of the church, which taught that his mental disturbance was the result of demoniac possession, and he was treated in accordance with this belief; using special rites of exorcism directed to the spirit, supplemented by corporal chastisement and mortification of the flesh by isolation and fasting. With the disappearance of the monasteries the insane began to accumulate in the towns and villages, and thus came within the cognizance of the law, which at first concerned itself only with the poor, because the well-to-do took care of their own unfortunates. This undertaking resulted in a very direct classification and disposition of the insane. Those who were demented and harmless were looked upon as paupers and sent to the almshouse; while the aggressive and violent ones were regarded as criminals and were sent to the gaol. The indifference of the insane to their personal welfare brought about another kind of classification, which resulted, among the almshouse population, in the separation of the cleanly from the filthy. The latter being like cattle, dependent upon others for their personal care, were herded in sheds littered with straw. In the gaol the classification followed the same simple lines. The quiet ones were treated as ordinary criminals, and those who were more or less continually disturbed were manacled, or chained to the wall or floor.

These, with slight variations, remained the means and method for the care of the insane until the period of the French Revolution. It is true, that, from time to time, efforts were made by individuals towards the amelioration of the condition of the insane, and a clearer and more rational conception of what was constituted in insanity, but without avail; for tradition and custom are stubborn things, and public enlightenment is not accomplished by revolution. However, as a result of the general upheaval and overturning which followed the revolution in France, an opportunity came for a demonstration that the insane were human and amenable to the same influences that affected the conduct of the sane. Almost simultaneously, in England, an effort was made by a member of a certain religious body to carry out in the care of the insane the tenets of his religious belief, and follow the precedent laid down in the New Testament, substituting quiet, cheerful surroundings and active personal care for the lash, ducking-stool and chains. The precedent established in England for the founding of York Retreat by the Society of Friends, through William Tuke, led to the establishment of other institutions using similar methods, and the influence of the teachings of Pinel and Esquirol, as well as the closer personal supervision of the daily life of the insane which resulted from this change of method, led to the recognition of the fact that the bodily condition of the insane man influenced his mental state; and, consequently, physicians began to be called upon to attend to the bodily ills of the patient. There had been no real change, however, in the conception of what was constituted in insanity, and the body and mind were still considered to be separate entities, possibly co-ordinate in action, but independent. Consequently, while steady progress was made in the amelioration of the conditions surrounding the insane man, and his clothing, housing and feeding were given more intelligent consideration, no effort was made toward the clinical study of the physical conditions present in insanity, nor were those incidentally observed in the study and care of the mental disturbance correlated with the varying manifestations of the insanity, and the degree of their interdependence in any way determined. On account of the former neglect of these unfortunate people, and the terrible nature of their surroundings, public attention and interest were concentrated upon the improvement of their personal care for a long time, and the economic necessities of the public institutions fully occupied the time of the medical men in charge. Besides, the insane were rarely brought to the public hospital until their condition had become chronic, so that there was little to be hoped from medical treatment applied to the restoration of mental capacity; while the establishment of these institutions in the country isolated the medical men in charge, and deprived them of the stimulus which comes from contact of men in the same work with each other. Consequently our public institutions were entirely occupied with the problems of personal care and economic administration, and there was no material advance in the methods of study towards the clinical rather than the custodial aspects of the work. It is true that during this time, as always, certain men tried to break the shackles of tradition and custom, but without avail, for the time was not ripe, and public opinion was not enlightened to the point of appreciation of insanity as a manifestation of disease. Singularly enough, the next great advance in the study of insanity came after the civil war in this country, and was a result of the stimulus of that great upheaval toward active effort in every direction. Besides, improved means of investigation had brought about a revolution in our methods of study of the nervous system; and its relation to the rest of the organism was beginning to be better understood. Through the efforts of men who made the study of nervous disease a specialty, both in this country and abroad, and as the result of their criticism, a more careful investigation of the pathology of insanity was begun. Then, too, the general medical profession began to take a more active interest in the study of insanity, its probable causes, pathology and treatment. Men doing special work saw in mental disturbance an apparent result or complication of diseased conditions which came under their care, and with which they were familiar, and each has tried to explain the manifestations of insanity from the standpoint of his special field. Among neurologists the effort is being made to explain the perversion of mental activity in terms of disease of the sensory and motor parts of the nervous system; while the medical men in the hospitals for the insane are still hampered by the inertia of custom and tradition, and the persistence of the metaphysical conception of what is constituted in insanity. These apparently indefinite methods of study have so far been barren of practical results. They are, however, tentative efforts in the right direction, because, in them all, there is the tacit recognition of the fact that the mind, so-called, is not an entity, and that its activity must necessarily be correlated with all of the organic processes. Reference to text-books on the subject of insanity will show that very little if any attention is given to the physical condition of the patient with relation to his insanity.

A great deal of space is given to the elaborate discussion of the manifestations of aberration; and the degree and form of insanity is the basis for its classification; while in the study of its pathology attention is given almost entirely to the morphology of the structure of the cortex. Again, the various manifestations of insanity are described as the specific result of some bodily disease or condition; such as fever, septicemia, traumatism, or the puerperal state; and even as the result of surgical procedure; entirely ignoring the fact that while all of these conditions affect the many, only the relatively few become insane; and that there is no correlation between the kind and extent of the physical disturbance and the form and persistence of the insanity. Indeed, when insanity accompanies or follows physical disease or traumatism, it is much more likely to result where these conditions are mild or slight.

The confusion resulting from the divergent methods followed in the study of insanity, and the varying conceptions of what is constituted in its manifestations, can not help but obscure the subject and make it difficult for the general practitioner of medicine to understand what he is dealing with, to recognize the early manifestations of insanity, and to carry out a method of treatment which will be rational and successful. The first step in the clearing up of this confusion is the recognition of the fact that probably no one ever becomes insane who is not primarily unstable or defective mentally! This instability or defect may be the result of hereditary conditions, or follow some cause of arrest in development, operating during the first seven years of life, during the period of rapid growth of the brain. Besides, it is not only the children of the insane who become insane, but, to quote the writer: "The recognition of hereditary influence has therefore to take into consideration the transmutation of form in the transmission from one generation to the other of constitutional or diathetic conditions affecting the parents, and even the effect of temporary causes affecting the vitality of the parents at the time of conception. That is, any condition which produces somatic degenerative change in the organism of the parents may be manifested by brain degeneration and mental aberration in the offspring. Thus the children of the syphilitic, tuberculous, or gouty, may be, and commonly are, the victims of degenerative disease of the nervous system and insanity. So that, while as generally understood the children of the victims of these diathetic conditions, who become insane, do not directly inherit the tendency, practically they do by the transmutation of the diathetic conditions between parent and child." We have to consider then in any given case of insanity, first, the personal history of the patient, for the indications of instability or defect in the nervous system, and its degree. Next, the family history; seeking for evidence of constitutional conditions which will indicate the cause of the limited cerebral potentiality in the patient, and enable us to determine its degree. Finally, we must study the actual physical condition of the patient, to determine the presence of disease or degeneration in the vegetative organs, which may account for the impaired vitality which has preceded or accompanied the outbreak or development of mental disturbance. It has been customary to consider that the excitement or depression manifested by an insane person was entirely independent of his bodily condition, and his activity and apparent indifference to physical discomfort have been considered as signs of good health. A few medical officers of hospitals for the insane used to go so far as to assert that the insane did not need any medical treatment. It is needless to say that such statements or practices were based on *a priori* reasoning, for a very little investigation will show that while the mental aberration is a manifestation of perversion in the functional activity of the brain, it is necessarily preceded and accompanied by some kind of physical disturbance, and in a great many cases has for its antecedent or concomitant grave organic disease of the body. At any rate, we have no right to say that a patient is free from disease until by careful examination, with the aid furnished by the laboratory, we have determined the facts. The mind not being an entity can not be treated directly. Also, as the nervous system supplies nothing toward its own nutrition, and is not concerned in the elimination of the waste materials of its own functional activity, but is, on the contrary, entirely dependent upon the vegetative organs for maintenance, it may be assumed, in consonance with current hypotheses concerning the role of auto-intoxication in the production of disease in the nervous system, that failure in the functional activity of those organs engaged in the reduction and elimination of waste material from the organism would have a more important bearing upon the development and persistence of perversion of functional activity in the general nervous system, as manifested by mental aberration, than would a similar failure in the function of those organs having to do with the preparation of food material for assimilation. In this connection, it must also be recognized, that, as it is the principal function of the general nervous system to direct the activities of the rest of the organism, a failure in this dirigent function would, of necessity, interfere with the normal activity of the vegetative organs, thus lowering the vitality of the whole organism, and further reducing its capacity for adaptation to external relations; by involving the retention, not only of the waste materials of its own activity, but also of such toxic substances as might result from the imperfect or incomplete conversion of the food materials taken into the digestive tract. It follows then, that in the treatment of insanity we are not directly interested in the mental condition of the patient. In my own experience I have never been able to see why an insane man should be treated differently from a sane man, who was ill and whose conduct made it necessary to substitute for his own will some form of external control. We recognize in physical illness and injury something that is tangible, that we can in a great measure understand and appreciate. Besides, unless delirium is present, neither of them interferes with our relations with the person who is ill or injured; whereas insanity first manifests itself by interfering with the relation of the patient to his surroundings; consequently we are alarmed, and feel helpless, because there is nothing apparent to account for the difference in character and conduct, which slowly or suddenly makes its appearance and changes the individual into an unsocial being, whose behavior is mortifying or alarming, and who must, in some way, be restrained; while he, in his turn, on account of the confusion and self-consciousness which characterize the beginning of the loss of mental capacity, sees in the manner and conduct of his relatives, and the consternation of his friends, full confirmation of the vague suspicion and unrest which have come to fill him with alarm; and in every word and action of theirs he sees something inimical to his welfare, while all that happens about him is distorted into suggestion of the disagreeable and untoward. For a long time he may be able to control himself, but, sooner or later, under the influence of fatigue, worry, or illness, the confusion will increase, and there will be a sudden outbreak of excitement or even violence. Instead, the picture may be reversed, and he will see all of the evil in himself instead of in his surroundings; become depressed, seeing nothing in the future but humiliation and defeat for himself, danger or disaster to his family. These are the people who suddenly commit suicide, or else first homicide and then suicide. These tragedies are of daily occurrence, and yet the relatives and friends of the victims, while they are cognizant of the changes in the character and conduct of the man, never think of attributing them to their real cause; because he converses and answers questions intelligently, and is apparently able to attend to his business; forgetting that in the presence of mental aberration his very intelligence makes him more dangerous to himself and others; because it enables him to guide and control his conduct in accordance with his perverted view of his surroundings. He, however, knows what his condition is, but tries to hide it even from himself, and would deny vehemently that there was anything the matter with him. And yet this is the time when the mental alienation is most amenable to treatment, and the prospect for permanent recovery the best.

To the well-to-do, who can supply the necessary conditions, the care of such a patient is not a problem; but to the ordinary family, especially when the individual affected is the bread-winner, the matter is very serious; and that is why, now that we know the importance and value of early treatment, that everything should be done, that can be, to educate public opinion to the appreciation of what is involved in insanity, so that means may be provided for its proper treatment, and the people educated to look upon insanity from the standpoint from which any other disease would be considered. But even the well-to-do are apt to lose sight of the most important element in the proper treatment of this stage of mental disturbance. That is, the substitution of some form of external control for the self-control that has been lost. This can never be successfully done by the members of the family of the patient. Their interest is stronger than their judgment, and their fear overcomes their own self-control; so that there results the spectacle of the will of the patient dominating the will of the care-takers, and the patient who can not control himself is allowed, and even encouraged, to do things which his relatives would not tolerate if he was well. In other words, the care-takers follow the lines of least resistance, and thus exaggerate the very conditions they are trying to remedy. It is only necessary to take into consideration the facts in the natural history of insanity, which explain the mental condition of the patient, in order to appreciate what is necessary to his proper treatment. But we must first disabuse ourselves of the conception which has resulted from the persistence of the picture of what insanity is after the development of mental reduction and complete loss of self-control. If we will remember that insanity, like every other disease condition, has a beginning; that it has to do with that part of the organism which exercises control over all of the vital processes and forms of activity; that the first manifestation of this impairment is confusion and loss of control, especially of those activities which are manifested in conduct, we shall appreciate what it is we have to deal with in its early treatment. However, we constantly see about us the mental conditions which are present in insanity. They are manifested as the result of acute alcoholism, of fear, anger, or mental overstrain, and they are frequently present as concomitants of disease, such as typhoid fever, septicemia, and meningitis. Therefore, they do not differ in kind from the ordinary manifestations of impaired vitality or toxemia, which result from general disease conditions. Consequently, we have to look elsewhere for the cause of their persistence after the subsidence of the acute disease process, and of their development without apparent physical cause. When we find some part of the organism easily affected by disease, or unable to bear its share of the organic division of labor, there is the natural presumption that it is incompetent from some cause; and if there is no history of previous disease, we are justified in the conclusion that the organ or part is congenitally unfit. No one questions this conclusion as applied to the vegetative organs, and yet they never think of applying it to the general nervous system, except in the presence of gross manifestations of defect. As the result of this method of study, insanity is attributed to disease, traumatism, overwork, etc., as if they directly produced the mental aberration, ignoring the fact that all of these conditions are present constantly and affect considerable numbers in the community, but it is only in the occasional case that mental disturbance is associated with them. Besides, there is no definite relation between the degree or nature of the physical disease or traumatism, and the form or character of the mental disturbance. We must, therefore, in the treatment of insanity, first take into consideration the cerebral potentiality of the individual, as shown by his natural capacity for self-control and persistent effort; and also consider the evidence furnished by his response to the ordinary conditions in his environment, of instability or defect. That is, is he as competent as those living under the same social and industrial conditions, in his community, to bear the stress and strain of competition, hardship or failure? If not, it is only necessary that some sufficient strain upon the vitality of the general organism should take place to cause his mental breakdown; to set up the disintegrative process that unfits the unstable brain for the performance of its functions and leads to their aberration. Consequently if we find these conditions present, the first thing to do is to put the brain at rest as completely as is practicable. This can only be done by controlling the conduct of the patient, and especially by changing the conditions in his environment which have been disastrous to his mental welfare. Then, bearing in mind the action and reaction between the general nervous system and the rest of the organism, we must establish such a régime as will result in the stimulation of elimination and the development of nutrition, to remove all sources of irritation, and restore the vitality of the nervous system. So that, after all, the treatment of insanity is just the same as the treatment of any other disease; excepting that we have superadded the necessity to overcome the loss of self-control, as manifested in the bizarre or untoward conduct of the patient. Only superstition and the persistence of medieval ideas as to what is constituted in insanity make us consider these natural manifestations of impaired vitality in the unstable nervous system to be the real disease, which we try to overcome by physical restraint or the use of drugs. We constantly lose sight of the fact that insanity is not an acute condition, self-limited, or to be terminated by surgical procedure; and that in its treatment time is the most important factor. Besides, like other chronic constitutional conditions, the result of defective organization, it is, in the majority of cases, but the expression of a progressive degenerative change. Usually this change has been so gradual as not to have attracted the attention of the family or relatives until some overt act, widely at variance with the ordinary conduct of the individual, rudely awakens them to the true significance of his condition.

As before stated, it is only the well-to-do who can supply the conditions in their own homes which are necessary to the proper treatment of the insane. The advantage the hospital is supposed to have over the private house is the greater facilities it offers and the more efficient nursing and care supposed to be given. You do not hesitate to take a person suffering from bodily disease to a general hospital to be treated, and this is being more and more commonly done, because the public has come to know that the systematic care and treatment received in the hospitals, where all of the appliances exist for such purposes, is much better and more apt to be successful than any care that can be given in an ordinary private house. How much more is this true in the care of the insane, where the very nature of the disease makes its manifestations interfere with the relation of the patient with his surroundings; and the changes in his character and conduct are based upon and influenced by beliefs concerning his relatives and friends. Besides, there is no disease condition where the properly trained nurse is so absolutely essential to success in the care and treatment of the patient. The whole art in the personal care of the insane man is to know how to let him alone; and if his treatment has been begun early enough, and carried out intelligently, he will seldom reach the state where physical restraint would become necessary. The behavior of the insane man is very much what is expected of him, and even after he has, in a great measure, lost his self-control, his conduct will be the reflex of his surroundings and the attitude of his care-takers toward him. So that, in the treatment of the mental aberration, more is dependent upon the trained intelligence of the care-takers of the patient than upon the conventional routine methods used in the treatment of disease. The noise the patient makes, the motions he goes through, or his conversation, have no significance with relation to the necessities of his medical treatment; although they are, in a measure, a guide to the proper supervision of his conduct. The clinical aspect of insanity is presented in the disturbance of the functions of the bodily organs of the patient, as the result of disease or degenerative change, and the conditions resulting, because they interfere with the nutrition of the brain, require active medical treatment. In their study and relief, when practicable, is comprised the medical treatment of insanity.—*Northwestern Lancet.*

---

### NEURASTHENIA.*

GENTLEMEN: I am able to bring before you this morning several examples of a nervous malady which gives rise to much distress of mind and body.

CASE 1. The first patient is a man fifty-one years of age. He has a look of strength and has formerly been a strong, hard-working man, but is now broken down, and his nerve-centers are debilitated. He is a carpenter by trade and has been ill for ten months, although he has lost little, if any, weight. For the period named he has been unable to do any work. About two years ago his child had a severe attack of diphtheria with complications and was sick for seventeen weeks. During all this time he was greatly worried. After the child's recovery he suffered from dysentery. This disease left him in a weakened state with

*Delivered by J. V. Shoemaker, M.D., LL.D., Professor of Materia Medica and Therapeutics and Clinical Medicine in the Medico-Chirurgical College of Philadelphia, in the Amphitheater of the Medico-Chirurgical Hospital.

shaken nerves. He was constantly restless and could not long remain in repose. He would walk about continually, and at night he would often rise from the bed, unable to sleep or even to rest. After a while his digestive powers failed and he was afflicted with flatulence. Next he began to have forebodings. If on a bridge or roof he felt an impulse to jump into the river or the street. His memory is good, but he finds that he has some trouble in counting, and he lacks concentration of mind. He is still restless at night. He has now no pain, but a general sense of weakness. He formerly suffered from pain in the chest, head and back. As a result of mental anxiety and physical ailments his nervous energy has been sapped. He is a wreck of his former self. There is no gross organic lesion of the central nervous system, but its functional activity is seriously depressed. The patient experiences no pain when I press rather forcibly upon the vertebral column. His heart is irritable and overactive. The pulse is correspondingly weak and tremulous. In many cases of this nature there is marked irritability along the spine. The man's tongue is flabby and white.

In this case we are dealing with a morbid condition due to diminished nutrition of the nerve-centers. This is known to-day under the name of neurasthenia. From the intimate relationship between the nervous system and the entire round of vital processes the symptoms of the disease are exceedingly varied.

CASE 2. Next comes a young woman twenty-nine years of age. At the age of twelve she had a depressed fracture and, so she states, was trephined. Not long since she had typhoid fever, which left her in a prostrated condition. Four years ago both her ovaries were removed. For seven months she has been troubled by sharp pains in the back, extending to the head. There has also been pain in the eyes and ears, although vision has not been disordered. At the present time distressing vomiting is the chief symptom of which she complains. The temperature is about normal. She is extremely anemic. The skin and mucous membrane are excessively pale. Her hair is dry and without luster. Her finger-nails are white. She is constipated. She is very nervous and tremulous. She vomits everything she eats and has sometimes vomited blood. There is no cough. Her stomach has been washed out three times. Her nose is pinched, her lips and tongue almost bloodless. She has pain at the pit of the stomach. The conjunctivæ are yellowish white. Her temperature is subject to rapid fluctuation. It rises and falls, but there is no constant fever. Her mind is very apprehensive. She has imagined she had nearly all the diseases in the category.

This case is not so plain for diagnosis as that of the man. We ask ourselves where is the starting-point of the chain of ills—in the blood, the secretions, or the nervous system? The stomach will be washed out again and its contents examined chemically and microscopically. A blood-count gave 3,200,000 red and 10,000 white corpuscles, with 65 per cent. of hemoglobin. As a result, however, of a careful study of this case I am of the opinion that the state of the blood is secondary to and dependent upon that of the nervous system.

CASE 3. The third patient is an older woman, thirty-three years of age. Her parents are healthy. She has had measles, diphtheria, typhoid fever, pneumonia and two attacks of nephritis; has undergone three major gynecological operations, including the removal of both ovaries—a list of afflictions which is certainly capable of accounting for diminished vitality and nervous force.

Three years ago, after taking a purgative, she had a profuse hemorrhage from the bowels, in consequence of which she was in a hospital for seven weeks.

For three weeks she has had pain in many parts of the body—"all over," is her own expression. One week ago the pain was practically located over the coccyx; it was of a dull and aching character. During the last three days it has predominated in the abdomen and near the heart. For the last three mornings also she has been troubled by nausea and vomiting. The pain was worse at night and prevented sleep. She is very excitable and nervous. The urine is of a specific gravity of 1,022, is acid, deposits a slight sediment, and contains a large amount of phosphates with some oxalates. The temperature is normal. The tongue is clean, but tremulous. This is evidently and without doubt a case of neurasthenia.

CASE 4. The fourth patient is a young woman aged twenty-two years. She is unmarried and a waitress by occupation. Four months ago she had an attack of vomiting upon the street. This was followed by a convulsion, which was regarded as hysterical by a medical man who saw her at the time. Four or five days later she had a similar attack, called heat exhaustion at the hospital to which she was taken. This was during a heated term. Since then there have been frequent attacks of vomiting and convulsions. The bowels have been very much constipated and there has been suppression of urine. In one of her paroxysms no urine had passed for twenty-four hours; she was catheterized and two quarts were withdrawn. Her temperature is irregular and often above or below the normal. To-day her tongue is tremulous. Her eye-lids quiver and tremors pass over her whole body. In this case we witness a mixture of neurasthenia and hysteria. The two pathological conditions are by no means identical, but are not infrequently associated in the same individual. This girl has a marked gastric catarrh as a basis of her nervous disorder. She is of unfortunate heredity. She is the daughter of a dissipated, perverted mother of nervous temperament. One of her brothers is anemic and nervous; another is a pervert. All the members of the family are self-willed and unruly. This girl often leaves her home and lives none knows how or where, but finds her way at last to some hospital.

From a study of these cases we learn many of the symptoms of neurasthenia, although, as you can easily understand, its manifestations are of a manifold character. The disease is, in many instances, due to inherited causes or neuropathic organization. It occurs in both sexes, and may be caused by overwork, either bodily or mental; anxiety; emotional shock; sexual disorders; and the debility induced by syphilis and other severe constitutional maladies. The victims of neurasthenia are easily fatigued by slight physical exertion, although they may present the semblance of health to the eye. A young man of whom I have knowledge, originally strong, has for more than a year been so weakened and timorous that he has given up his occupation and does nothing but sit about the house, a prey to all sorts of apprehensions. Lack of ability to concentrate the attention is common. These people are irritable and excitable. Headache, vertigo, and insomnia are frequent complaints. The senses of sight and hearing are not infrequently affected. Tremor is another symptom which is often observed in neurasthenics. The reflexes are exaggerated. There is apt to be excitability and palpitation of the heart. Not uncommonly there is irritability of the bladder. Digestive disturbances are likewise prominent. The appetite shows a tendency to capriciousness; there are pain in the stomach, constipation, and various signs of indigestion. Paresthesia and hyperhidrosis are not unusual features of the disease. Phosphates and oxalates appear in the urine. In uncomplicated neurasthenia the convulsions, paralyses, and stigmata of hysteria do not occur. In not a few instances neurasthenia and hypochondria are conjoined.

Many of the manifestations which I have thus cursorily outlined are exemplified in the patients shown you to-day. The variations of the general picture, however, are almost infinite. Certain features predominate in some cases, while in others we will observe quite a different symptomatology. Nevertheless a careful analysis will convince us that they all depend upon a depressed condition of nerve-centers. This is the key to the most diverse phenomena.

The *treatment* of neurasthenia must be conducted upon general principles. We must seek to identify the cause which in each case has given rise to the lamentable condition in which the patient is plunged. Rational rest will accomplish much, and by this I do not mean absolute idleness. If a case has originated in physical strain and hard work the hours of labor should be shortened or lighter and less exacting occupations should be substituted. In the larger proportion of cases, however, it is excitement or unrest of the mental and emotional natures which have led to the affection. In young people it is very apt to arise from sexual aberration or excess and from emotional shocks. We should endeavor to promote and encourage the formation of totally different habits of life. The hurry, the bustle, the whirl of business competition should be abandoned and the patient should be directed to adopt and cultivate more simple habits and cast aside anxiety. This is, doubtless, a hard prescription to follow. One of the most effective methods of combining leisure with variety and interest is travel. A journey has a strong tendency to divert mental activity into healthy channels. Morbid tension is diminished, irritability is decreased, the tone of mind becomes more cheerful, and with this improvement the functions of the entire nervous system are strengthened. An ocean trip will often produce a very decided improvement or even a complete cure. As an instance, I may refer to the case of a burly Scot, who, in consequence of the cares and anxieties of a large business, had become neurasthenic. He had received little or no benefit from purely medicinal treatment and was advised to take a long sea voyage. He did so, and in the course of his travels visited his native country. The result was all that could be desired. Seasickness occasioned a strong revulsion. When this subsided the appetite and digestion were markedly improved. The sight of the spots where his youth was passed, the welcome of old friends, brought about a happy renovation of body and mind. His worry and brooding disappeared, and he returned home a well man, completely cured. I can recall other cases in which the result of a sea voyage was no less conspicuous. The influence of the change of climatic conditions is analogous to and often allied with the advantages of travel. Life in the open air in a salubrious climate is remarkably efficacious in restoring the tone and power of the nervous system.

Electricity is often of value. I am much in the habit of using strong galvanic currents—40 to 50 milliamperes—along the spinal area and over the abdominal viscera. We may begin with a weaker current and gradually increase the strength according to toleration and effects. The usual effect of such a procedure is to augment the nutrition, quiet morbid excitability, and improve the digestive power. The diet of neurasthenic patients demands care. In aggravated cases a milk diet for a certain period is the most advisable. After a time we may enlarge the list by the addition of soft-boiled eggs, rice, baked potatoes, beef, and lamb. Massage is an excellent adjuvant to electricity. The manipulations should be performed by one who is familiar with the art in all its methods. In this connection I repeat what I have often said to you of the importance of acquainting yourselves practically with the manipulations of massage.

Drugs are of less value than these methods of hygienic or physiological treatment, to which I may add the systematic and rational use of hydrotherapy. Cold rubs and wet packs do good by rousing the activity of the circulation, increasing nervous energy, and promoting sleep, which is so often fitful and broken in this disease. Such means are decidedly preferable to the use of coal-tar products, hypnotics, and sedatives. Although these are largely employed and sometimes accomplish temporary good, yet they are of no permanent avail, and, in fact, are capable of doing injury. Nor is it wise to be continually dosing the patient with medicines. The effect may be to cause additional impairment of an already weakened digestion or to establish a drug habit; in either instance the patient's condition will be worse than the first state. Let the drugs which you prescribe be of such character as to lend strength and support to the nervous system. Arsenic, phosphorus, iron, zinc and sometimes an antispasmodic may do good in selected cases or at certain times.

In those cases frequently, especially among young men, where neurasthenia is dependent upon irritation of the sexual apparatus consequent upon solitary vice or excessive venery, the local application of electricity is of more potency than any other form of treatment. Of this branch of the subject I shall doubtless have an opportunity of speaking on some future occasion. Unhappy sufferers from sexual nurasthenia are numerous and are constantly applying at our clinics for relief.

Now, to apply these general principles to the individual patients here this morning:

In the first case, that of the man, I shall employ galvanic electricity in the manner described, the anode over the spine and cathode along the abdomen. As his condition has been induced by the natural anxiety of a parent combined with the sleeplessness and physical wear incidental to watching by a sick-bed, and as he does not belong to the class addicted to introspection and self-analysis, I have little doubt that he will eventually recover his strength and again be able to work at his trade. For medicine he shall have the solution of arsenous acid—liquor acidi arsenosi—in ascending doses until signs of its physiological action are observed, when it shall be discontinued for a few days, to be resumed in small doses and again gradually increased in the same manner. Arsenic is one of our most reliable pharmaceutical remedies in neurasthenia.

The second patient shall be ordered a suitable amount of rest and shall be given silver nitrate in ¼-grain doses three times a day. This drug has a beneficial influence upon the gastro-intestinal mucous membrane and also upon the nerve-centers. It will promote nutrition and stimulate peristalsis and elimination.

In the third case we shall begin the treatment with massage and subsequently give static electricity up and down the spine. As an antispasmodic and nerve tonic she shall be given:—

℞ Zinci phosphidi........................................gr. 1-10.
Extracti sumbul........................................gr. j.
Sodii arsenatis........................................gr. 1-20.
Zinci valerianatis........................................gr. j.
M. et ft. pil. No. j.
Sig: One such pill three times a day.

The fourth case offers little promise. The poor girl is a degenerate. She is treated symptomatically in the hospitals from time to time for special outbreaks, but in the intervals lives as she pleases and is totally without medical treatment or moral guidance. It is difficult to treat such an individual with any prospect of success.—*Medical Bulletin.*

---

### BUSINESS METHODS FOR A PHYSICIAN.

BY C. U. COLLINS, M.D., PEORIA, ILL.

When the writer was a lad just entering into the study of medicine, Dr. A. and Dr. B. were the leading physicians of the town in which he lived. Dr. A. had two distinguishing characteristics. He answered calls promptly and he kept his books posted to date. He was a good collector. Dr. B. was always several months behind in the posting of his books. The writer once saw a man go from the bank to Dr. B.'s office with a roll of bills to settle his account and the doctor told him to call again, as his books were not posted.

This seemed at the time little short of criminal and still seems so. The writer made a vow then that no man who wanted to pay a bill should be turned away from his office, and the vow has been faithfully kept.

Laying aside all sentiment concerning the practice of medicine that has been handed down from the past when physicians made no charge but accepted honorariums, the fact remains that the physician, like the man in business, must keep his income equal to or above his expenditures in order to maintain his credit. The business men from whom he buys expect him to be able to pay for what he buys; so there is a business side to the profession of medicine and we can not get away from this fact.

If he collects a goodly amount each year, it not only enables him to keep his family comfortably, but it also enables him to buy books, instruments and other accessories that make it possible for him to render better service to his patients, and this is a very good argument to give to his patients for collecting promptly.

With regard to collections, physicians can usually be divided into two classes. One class answers all calls without regard to payment. They act on the theory that their buggy being seen on the streets and before the doors of patients is an advertisement. They make no systematic, determined effort at collecting for fear of making some one mad. They never try by legal process to force a delinquent patient to pay his bill. They aim to be always busy, therefore they never refuse a call, although the patient may have made no effort to make a payment on his account for years. This class has very little time for study, and they only collect about fifty per cent. of their charges, thus doing two dollars' worth of service for one dollar. Yet they often have enough income to more than pay their expenses and are successful in a sense.

Physicians of the second class endeavor to collect from all who are able to pay, even invoking the aid of the law if necessary. They would rather do less business at a proper rate of remuneration than double the business at half pay. Their income will be the same and they will have time for study and research into the problems of their profession. They will render charity as quickly as any one, but if the patient is *able* to pay, he is expected to pay, even if it is only a little at a time. They know that a physician's worst enemies are those who have old unsettled accounts on their books, and these enemies can be changed into friends if they can only be made to settle their accounts.

The writer has endeavored to belong to the second class, believing that it held the best opportunity for professional and financial success.

Granting that there is a business side to the profession of medicine, the next question is, how can that side be best developed? It is very necessary that the physician have a system in conducting his business. He must have a good system for keeping his accounts. It is not necessary in this article to call attention to any particular system, but there are a number of good systems, designed especially for the wants of a physician, on the market. The account should show the correct name and address of the head of the family, with any other information serving to identify him, as, for instance, the place where he works. It should show what the service was and to which member of the family it was rendered, as well as the date and charge. These little details facilitate the collecting of an account wonderfully.

The physician should consult an attorney and post himself regarding the law governing collections in the State in which he is practicing. He should ascertain how many years an account can run before it is outlawed, how much property is allowed a debtor under a schedule, and how much wages under a garnishee. Incidentally he should find out whether the particular system he has chosen for the keeping of his accounts will stand the test in the courts of his State. Some systems are faulty in this respect. The books should be kept posted to date, so that any debtor who wants to settle his account can be accommodated without delay.

Statements of account should be rendered to patients at certain regular intervals. The physician can determine the best time for sending these statements. He can choose the intervals best adapted for his practice. Some physicians of the writer's acquaintance find that three times or twice a year is best suited to their locality.

The writer has held that a medical account differed in some respects from a mercantile account. The patient is generally forced to contract a medical bill whether he is prepared for it financially or not; while he can put off the contracting of a mercantile account, usually, until he has some prospects for meeting the obligation. Therefore, while it is all right to send a statement of a mercantile account the first of each month, it might not be wise to send the statement of a medical account so soon or so often. It is apt to be burdensome to the physician to go over his books and send statements of accounts twelve times a year, and if the patient receives the statement too often its moral effect is lost.

Personally, the writer has thought that four times a year, or the first of each quarter-year was the best time for sending statements. His statements bear this message :

"It is my custom to present statements of all accounts on the first day of January, April, July and October. To avoid misunderstandings and to correct mistakes, should any occur, you will please call upon me at the office at once if this statement is not satisfactory. The items of this account can be examined at the office. It is expected that at least a payment will be made on this statement before the next one is presented."

By sending the statements at certain stated times the patient knows when it is coming and can prepare for it. If he is sensitive about receiving a statement he can pay the bill before the time comes for sending it. The writer's collections are always good for two weeks before the first of the quarter. By this method the patients know there are four definite dates during the year at which they are expected to notice the account, either by paying it or by making some explanation concerning their inability to pay it.

A memorandum of each statement sent is made on the ledger page, giving the date and amount. If there has been no response when the second statement is sent the last sentence of the message is underscored, and a few discreet inquiries are made of "the butcher, the baker and the candlestick-maker," or grocer regarding the patient's promptness in paying his bills. In this way only six months' service is rendered before the patient's reputation for paying is known. If he comes in and "has no money, but a good excuse," he is asked to set a time that he can pay. This time is marked on the ledger page bearing his account while he is present, and the promise treated seriously, as though it was earnestly expected that he would appear at the given date. If he sets the time for paying more than six months ahead, he is asked to sign a note for the amount due, drawing interest at the legal rate. A definite time for settlement is thus set and this is the secret of good collections. If the patient proves to be a professional dead-beat, no more service is rendered until he settles.

If he does not come to see about the account some method best suited to his case is instituted to force its collection. Two or three letters may be written, the account may be given to a collection agency or an attorney, or suit may be brought at once. There is one thing important in this connection. The physician must keep his word with the debtor. For instance, if he intimates that if the debtor does not come in to see about the account by a certain date he will bring suit at once, then the suit must be brought if the debtor fails to appear at that date. The writer has spent more money sometimes in collecting an account than he received, but the moral effect of the successful collection was worth a great deal more than it cost.

If collections are conducted for three or four years along these lines they will be easy from then on. The people will say, "Dr. Blank is a good doctor, but he expects his money." A reputation of that kind is worth thousands of dollars to any doctor.

Physicians are accustomed to bear the burdens of others and allow themselves to be imposed on many times. It often happens that a poor patient has wealthy relatives, who at the time of an accident or during a critical illness will tell the doctor to "go ahead and do all you can for him and I will see that you are paid." In law this statement is not worth the breath it took to utter it, and if the relative chooses to disregard it after the emergency has passed (and he often does) there is no way of making him keep his agreement. The writer could fill several pages with incidents where the wealthy relatives and corporations and contractors have refused to pay a bill after the emergency has passed, although agreeing verbally to do so before. He at last became tired of it and devised the following card:

Peoria, Ill., ________________, 190__

Dr. C. U. Collins:

You will please attend ____________________

________________________________ during his present illness.

____________________

Now, when some responsible party says, "go ahead," he is handed that card to sign, and then something peculiar always happens. Invariably this inquiry is made: "Well, doctor, how much is the bill going to be?" Under a verbal order, which the party seems to realize is not binding, no inquiry is made about the amount of the bill, but when he is asked to sign his name to the order-card he knows at once that that is business, and immediately wants to know how much he will probably have to pay. The writer has had a wealthy man absolutely refuse to pay a doctor bill for a step-son until shown an order-card signed by his wife. He would allow a hard working doctor to labor over a difficult case without any remuneration until he was forced to help bear the burden. A wealthy farmer once refused to pay a bill because his son was a month or two past twenty-one when the service was rendered, although he was very solicitous at the time of emergency and urged the doctor to do all he could.

In conclusion, the reader must not get the idea that this article recommends the doctor to be hard and grasping in his dealings with patients. The doctor can always be depended upon to take care of the widow and orphans, as he always has in the past. If a patient is unable to pay and there is no one else who should pay it for him, the doctor should and will attend him as faithfully as he would a millionaire, but it is necessary to adopt some business methods that will prevent imposition by those who are able to pay. —*Medical Standard.*

---

### MANAGEMENT OF PREGNANCY.

BY W. S. PHILLIPS, M.D., BELLE CENTER, OHIO.

The physician who conducts a case of pregnancy to a successful termination, in which, as is often the case, unaided nature is not equal to the emergency, has well earned the gratitude of one family and rendered a valuable service to the State.

For want of time we are obliged to refer cases of pre-existing or intercurrent disease on the part of the mother to the physician in the ordinary routine of his work, and to the abdominal surgeon, confining our paper to the morbid conditions growing out of the pregnant state, which annoy and menace the life of both the mother and her unborn child.

As germane to the subject, it is necessary to invite the confidence of the family in this matter of interest, in order that the physician may have his case well in hand from the beginning, as many cases terminating disastrously are due to lack of intelligent supervision, arising from want of mutual confidence. The physician who would render the best service to prospective parents should instruct his clientele as to the importance of intelligent supervision, and thereby secure their cooperation.

While pregnancy is strictly a physiologic process, human environment and habits of life render it one beset with danger to health and to life itself. When we have learned to pilot these cases to a successful issue, we are prepared to offer one of our best gifts to humanity.

Much has been written in the way of speculation with reference to stamping certain mental and moral characteristics upon the unborn child through the conduct of the expectant mother during the period of pregnancy. It is the belief of the writer that the inheritance of heredity is transmitted along the lines of what people are rather than what they may presume to be for a period of a few short months.

The first question to be settled in each case is whether or not pregnancy exists. An opinion is to be formed from the totality of signs and symptoms with which all are more or less familiar, but in any case of doubt, which at times occurs even in the minds of the most expert, it should be remembered that error must be made on the side of safety.

In the first place, it is good practice when meeting with any trouble whose origin may reasonably be referred to local causes, and which does not readily respond to hygienic and medicinal treatment, to make an examination with reference to the position of the uterus. Versions and flexions, especially when development causes impingement of the uterine wall against the osseous framework of the pelvis, give rise to both local and general disturbance and an eventual miscarriage. When no adhesions exist, these can ordinarily be remedied by reposition once or oftener if need be, maintaining in the intervals the degree of correction first gained by an easy-fitting support.

The next consideration, having especial reference to the welfare of the fetus, is the detection and correction of malpositions during the period of intra-uterine life. This is an especially serious matter, in the experience of the primipara during the last month of gestation, if the position of the child should be oblique or transverse. When the abdominal walls are thin, the malposition can frequently be detected and correction brought about by external manipulation, the correction being maintained by a snug-fitting bandage. However, if the usual longitudinal position be reversed, resulting in a breech presentation, detection is not so easy, and correction is difficult, if not impossible, on account of the usually scant amniotic fluid, with consequent short lateral dimensions of the uterine body, and it is better referred to the obstetrician well trained in the dexterous application of the forceps to the after-coming head.

The catalogue of diseases affecting primarily the expectant mother and directly traceable to gestation may, as a rule, be grouped into a single class, having their origin in the accumulation of effete products in the system, or in the disturbed balance between food ingestion, quickened processes of nutrition, and elimination of waste from the body.

That the nourishing of two similar lives in one body brings about any physiologic process essentially different from the ordinary, save in that of heightened activity, is incomprehensible; hence we conclude that the diseases having their origin in the pregnant state owe their existence to this circumstance. This conclusion is emphasized by the happy results of treatment based upon this view of the case. A single exception may be noted, that of nausea, which seems to be of mixed origin: first, reflex; secondly, toxic; and thirdly, a combination of the two.

The reflexes from the gravid uterus exert but a slight influence upon the other morbid states, except that of general nervousness, and consist of neuralgias, principally headache and enteralgia, vertigo, anasarca, eclampsia, insanity, pernicuous anemia, and tuberculosis. The first three mentioned are danger-signals to warn the alert physician of possible disaster if unheeded, and are the lesser manifestations of toxemia, first, upon the nervous system, and secondly, upon the fluidity of the blood, allowing extravasation into the lymph spaces and surrounding cellular tissue, thus relieving the blood of a certain amount of its toxicity. A coincident warning oftentimes occurs in the filtering of albumin through the overworked and irritated kidney.

While it is generally conceded that eclampsia is of toxic origin, it may also be assumed that insanity and pernicuous anemia, having their origin in pregnancy, are due to the same cause, for the reason that they are prone to disappear when the period of lactation is ended, and like insanity, having its origin in other conditions, have a predilection for the time of year when the average temperature is relatively high. In addition to an excess of chemic products, evidence is not wanting which indicates the presence of unused organic matter in the circulation, which we believe forms the basis of susceptibility to tubercular infection.

In the case of the healthy pregnant woman, observation reveals three of the physiologic functions noticeably altered, viz., appetite quickened, perspiration diminished, and exhaled breath unusually fetid, the reason for which is obvious. In the first place, the requirement for an additional food supply by the development of a new life, and the derivative effect upon the circulation due to the same cause, depleting the cutaneous vessels, renders the glandular structures of the skin functionless and forces the lungs to perform compensatory work.

That these alterations in the physiologic functions of the body in the case of pregnancy bear an important relation to the morbid states enumerated there can be little doubt. Some people inherit the inability to eliminate the waste products formed by the processes of nutrition, while in others it is acquired by disease in early life, and any circumstance calling for an additional food supply disturbs the normal balance between health and disease.

With this view of the etiology, it is evident that the management instituted to steer clear of these formidable complications must be dietetic and eliminative, and has been found successful in its empiric application during the lifetime of the present generation. The first principle, then, to be impressed upon the mind of the expectant mother is self-mastery. Appetite, whether natural or acquired, is the most tyrannical master in the sphere of the moral universe, and it is a fact that civilization is exemplifying the accusation recorded against Catiline, of "bowing his head like the beasts of the field in obedience to his stomach." Under no circumstances does self-control in this respect become more imperative than in pregnancy, and the physician should recognize the fact that there is a definite relation between the amount of food ingested and the excreta thrown off from the body, through the different channels of elimination; conditions of atmospheric temperature and exercise should be taken into account, and physicians should see that the amount of food eaten is commensurate with the ability of the excretory functions to take care of the waste material. Sometimes it will be found necessary, in order to have the patient properly nourished, to keep the skin, kidneys and bowels stimulated to activity by the different agencies at our command. Within reasonable limits the size of a babe at birth, as compared to that of its parents, bears no relation to longevity or mental and physical development in after-life; and while we may admit that a bountiful menu, generously partaken of, three times a day or oftener, as an essential ingredient in the ability to nurture and bring forth a bouncing baby of immense avoirdupois, it is a fact of no special virtue other than tickling the vanity of a proud father, while piled up in the other side of the balance is discomfort and disease, lack of muscular tone, making labor tedious, injury to some of the bodily structures which the skillful hand of the surgeon can only approximate to their original integrity, anguish of soul and body, and too frequently death, either before or after delivery.

The remaining perplexity with which one has to deal occasionally is reflex nausea, which does not yield to dietetic or medicinal means, and which has to be overcome by cervical dilatation, a remedy ordinarily efficient, and yet attended with grave danger to the life of the fetus. No definite rule can be laid down as to the amount of force to be applied or the extent of dilatation to be made, the toleration of the tissues of the mother being variable. A safe proceeding is to begin with the smallest amount of dilatation observable, using a steel dilator, increasing the amount of dilatation at intervals determined by the length of time required for the disturbance occasioned thereby to subside, until the trouble disappears or until the pregnancy is terminated.

An opiate administered hypodermically is a useful adjunct, as long as any hope is entertained of preserving the life of the fetus intact.—*Medical Age.*

---

### ORGANIC IRON MEDICATION IN SECONDARY ANEMIAS.*

A great deal has been written in recent years on the value of the various new organic iron compounds in the treatment of anemia, and our only excuse for the presentation of this report is that every new series of clinical observations, made with due conservatism and accurately recorded, is of value in confirming and disproving some fact or theory in medicine.

The problem of treating secondary anemias is an interesting one. In each case there is, in the first place, the primary factor, be it loss of blood through hemorrhage, spontaneous or traumatic; or be it the lowering of the functional activity of the blood-forming organs wrought by disease somewhere in the body, or by the action of toxins; or the direct destruction of the red cells and their hemoglobin in the circulating blood by some more violent toxic agency.

The first question, therefore, is how to remove the primary factor, or, at least, how to arrest its influence on the state of the blood. The second is, how to improve the state of the blood, so

*A Clinical and Hematological study by Lino S. Chibas, M.D., Senior Assistant House Physician, Columbus Hospital, New York, and G. A. De Santos Saxe, M.D., Assistant Pathologist to the Columbus Hospital, New York.

as to give it a new lease of life by increasing the amount of hemoglobin—that prime agent of oxygen exchange—and the number of red cells, the carriers of this agent.

In each individual case of secondary anemia there are different obstacles to be overcome as regards the primary factor, and therefore the treatment of the primary disease varies; but the therapy of the secondary condition is alike in all cases. Iron and its assistant, manganese, are the specifics to which we must have recourse—of that there has long since been no doubt—but the form of iron that should be used for this purpose is another question.

The problem as to the exact site and mode of absorption of iron which is administered therapeutically has occupied pharmacologists for a number of years, and a great deal has been written on the subject, and yet, there is still no agreement even as regards some of the essential points of this question. Is iron absorbed at all in the inorganic state? If so, in what form and in what quantities? What form of iron is most readily absorbed? How does iron act if it is not absorbed, or if only infinitesimal amounts, totally inadequate for the needs of the body, enter the plasma and are taken up by the molecules of hemoglobin? All these questions have been discussed and rediscussed, but as yet, as Hammarsten[1] says: "The action of the iron salts is obscure."

In a clinical article we are not called upon to go into details in discussing the various phases of the question as to the absorption and mode of action of the iron salts, but a few words may be said to show the present status of the subject.

Whether iron compounds of the inorganic group are absorbed at all, is a question of subsidiary interest in the present inquiry. There are two diametrically opposite views on this question. Bunge and his pupils[2] say that inorganic iron salts are not absorbed in any amount, however small, and that Blaud's pills and similar preparations act only by combining with the hydrogen sulphide and the alkaline sulphides of the intestines, thus preventing the decomposition of the organic compounds of iron existing in our food, especially in vegetables, and so permitting the absorption of these compounds into the blood. The opposite view is held by Quincke[3] and others, but the balance of evidence is in favor of

Bunge's hypothesis.[4] The well-known fact that enormous doses of iron are required to produce appreciable effects in chlorosis supports this theory. Thus, if a woman takes six grains of reduced iron three times a day (eighteen grains daily), it will take weeks to restore her to the normal condition if her hemoglobin has fallen to 50 per cent. And yet, the entire amount of iron in the blood of a normal woman of average weight is only thirty grains, so that if the inorganic iron were absorbed, as some observers claim, a few days would suffice to restore the balance of hemoglobin and red-cells.

On the other hand, organic iron compounds, especially such as are composed of iron with a proteid substance that resembles as closely as possible the proteids of the food as they occur in the intestine (*e. g.*, peptones), are undoubtedly absorbed into the blood in sufficient amounts to produce a comparatively speedy therapeutic effect in anemia, without injuring, as the inorganic compounds often do, the epithelial covering of the stomach and intestines, and thus causing gastro-intestinal symptoms summarized under the two general headings of dyspepsia and constipation.

It is these advantages that led to the general adoption of the iron peptonates, albuminates, etc., as the remedies to be preferred in the treatment of anemia. In this report we deal with one of these preparations, that known as pepto-mangan, Gude, in which iron and manganese exist in the form of peptonates. Gude's pepto-mangan has been used for a long time at the Columbus Hospital as a matter of routine in all anemic patients during convalescence from prolonged illness or from operations. The satisfactory results which have been obtained with this preparation have been noted, in a general way, by the visiting staff as well as by the house physicians, but until now we had made no study of the exact results, as attested by the examination of the blood before and after the initiation of the treatment.

In order to determine more accurately what could be expected of pepto-mangan in secondary anemias as they occur in a general hospital, we studied a number of cases in the medical, surgical and gynecological wards. Of these a majority were in the services of Drs. Ramon Guiteras and Egbert H. Grandin, visiting surgeon and visiting gynecologist to the hospital, and take this opportunity to acknowledge their courtesy in permitting us to pursue this work.

About forty cases were studied from October 1, 1902, to March 1, 1903, in as thorough manner as possible, with a view of determining the action of the preparation to be tested. Unfortunately, for reasons beyond our control, a great many of these patients left the hospital, believing themselves sufficiently improved, without giving us time to try the remedy for a sufficient period to obtain definite results. We present, however, twelve cases in which the medication was continued for three or more weeks, usually for about a month in each instance. In each of these cases blood-counts were made before beginning the treatment, as well as after it had been discontinued. The cases are given below, simply as they appeared in our notes, and they were not selected particularly on account of the results noted, but merely because they were the cases studied more completely than the rest.

### REPORT OF CASES.

CASE 1. Mrs. R. F., Italian, forty-two years of age, was admitted to the hospital on December 4th. Diagnosis, ovarian cyst. Symptoms of secondary anemia. She was operated upon December 5th and the uterus was removed through the abdominal incision, as it was found to be the seat of a fibroid tumor which had degenerated into sarcoma. She was discharged cured on January 10, 1903. During her convalescence she took one tablespoonful of pepto-mangan (Gude) three times daily. The examination of the blood showed the following findings:

December 4, hemoglobin 50 per cent., reds 3,350,000, whites 15,000. December 18, after hysterectomy, hemoglobin 39 per cent., reds 2,300,000, whites 16,000. January 10, hemoglobin 70 per cent., reds 4,250,000, whites 7,800.

The patient left the hospital in an excellent condition, showing no signs of anemia or debility.

CASE 2. A. P., Italian, twenty-five years old, admitted November 17th, with stricture of the urethra and signs of marked anemia. November 24th, perineal section and internal urethrotomy for stricture. There was considerable hemorrhage during and for a few days after the operation.

Examination of blood: December 12, eighteen days after operation, hemoglobin 68 per cent., reds 3,700,000, whites 10,429. January 4, 1903, twenty-eight days after beginning the use of pepto-mangan, hemoglobin 95 per cent., reds 4,800,000, whites 8,400.

Pepto-mangan was given in doses of one tablespoonful three times daily from December 13th to January 10th. The patient was discharged cured on January 10th, in good general condition.

CASE 3. M. S., Italian, twenty-five years old, admitted October 14th. The diagnosis was perinephritic abscess and tuberculous knee-joint, and the patient showed pallor of the skin and mucous membranes. He was operated upon by lumbar incision for perinephritic abscess on October 24th, and his knee-joint was excised December 18th.

Examination of blood: December 13, 1902, three weeks after first operation, hemoglobin 70 per cent., reds 3,104,000, whites 5,888. December 20, 1902, two days after excision of joint, hemoglobin 70 per cent., reds 2,750,000, whites 24,000. January, 10th, when discharged, hemoglobin 85 per cent., reds 4,640,000, whites 5,150.

This patient was given pepto-mangan for three weeks from December 21st to January 10th. He was discharged improved in general health. The anemia was very marked on December 20th after the second operation, and the increase in the blood-cells and hemoglobin was very satisfactory for a case of this severity after three weeks' treatment.

CASE 4. Ida M., five years old, Italian parents, born in the United States, was admitted November 30, 1902, suffering from typhoid fever. December 12th, after the convalescence had set in, the child was extremely anemic-looking, with pale skin and pale, bluish-red mucous membranes. Pepto-mangan was ordered, a teaspoonful three times daily, on December 12th. Eight days later the first blood examination was made, two weeks later, the second The findings of the pathologist were as follows:

December 20th, hemoglobin 75 per cent., reds 4,720,000, whites 30,000. January 8th, hemoglobin 85 per cent., reds 4,960,000, whites 9,200. The patient was discharged cured on January 8th.

CASE 5. Cesare C., aged twenty-five years, single. Had been operated upon one year ago in South America for vesical calculus and urethral stricture. Was admitted December 3, 1902, complaining of inability to urinate and continuous dribbling of urine through a suprapubic fistula. December 13th, perineal section without a guide and internal urethrotomy were performed. The patient was weak and anemic after the operation, so pepto-mangan, a tablespoonful three times daily, was prescribed on February 5, 1903. He made a good recovery from the perineal operation, but the supra-pubic fistula persisted. After twenty-two days' treatment with pepto-mangan he was discharged improved.

Examination of blood: February 6, 1903, hemoglobin 80 per cent., reds 3,878,000, whites 4,250. February 28, 1903, hemoglobin 85 per cent., reds 4,516,000, whites 4,600.

CASE 6. M. C., aged forty-four years, widower, has had urethritis four times. On admission he gave a history of having suffered from frequent and painful micturition for fifteen months. An examination showed a chronic urethral discharge, a urethral stricture, 12 F. at about 6½ inches from the meatus, and a tumor in the right umbilical region simulating a very large kidney. The prostate was much enlarged and very tender. The urine was of a specific gravity of 1,020, acid in reaction, contained no sugar and no albumin, but numerous pus-cells. In addition to treatment by irrigations and by dilatation of his stricture, he received pepto-mangan, a tablespoonful three times daily, from February 4th to February 28th, to combat a marked anemia.

Examination of the blood: February 5th, hemoglobin 45 per cent., reds 2,149,000, whites 9,760. February 28th, hemoglobin 55 per cent., reds 2,460,000, whites 6,890.

The patient improved as regards his urinary symptoms, but his anemia did not show much amelioration after twenty-three days of iron therapy. At the time of writing he was to be prepared for a second operation, an exploratory nephrotomy for his renal tumor.

CASE 7. A. B., Italian, aged fifty-eight years, married, was admitted to the hospital on November 24, 1902, complaining of symptoms of enlarged prostate which had been giving trouble for six months. He had lost considerable flesh and strength and looked very anemic. He was operated upon December 27th. His convalescence progressed satisfactorily as regards his urinary symptoms, but the anemia persisted, and on January 14th he was put on a tablespoonful of pepto-mangan three times daily After twenty-five days of this treatment he was discharged somewhat improved as regards the anemia. The report of the two blood examinations before and after the use of pepto-mangan was as follows:

January 15, 1903, hemoglobin 55 per cent., reds 2,940,000, whites 8,300. February 9, 1903, hemoglobin 65 per cent., reds 3,110,000, whites 8,100.

CASE 8. A. D., eight years old, schoolgirl, on admission to the hospital, September 22, 1902, complained chiefly of abdominal pain, general weakness, and enlargement of the abdomen. On September 24th the abdomen was opened, and the peritoneal cavity found to contain a large number of tuberculous foci on the peritoneum and a considerable amount of serous fluid. The diagnosis of tuberculous peritonitis was made.

On January 27, 1903, the abdomen was again found full of fluid, and was opened for the second time. On January 28th the patient was given pepto-mangan, two teaspoonfuls three times daily, for twenty-nine days, at the end of which time she was discharged. The anemia had not improved. The reports of the blood examinations were as follows:

January 29, 1903, hemoglobin 75 per cent., reds 3,920,000, whites 10,000. February 27, 1903, hemoglobin 75 per cent., reds 3,890,000, whites 7,200.

CASE 9. G. P., Italian, twenty-eight years old, was admitted to the hospital on January 13, 1903. For the last four months he had noticed a swelling of the left testicle. He had his scrotum tapped ten days before admission, and about five ounces of a clear fluid had been withdrawn. An examination showed a pyriform swelling about eight times larger than the normal testicle, with an apex above the external ring. Its upper part was hard, without fluctuation, dull on percussion, no impulse on coughing and non-translucent. Its lower part fluctuated and was translucent. On January 19, 1903, the testicle was removed, the diagnosis of sarcoma of the testis being afterwards confirmed by microscopical examination. On February 1st the patient was given pepto-mangan in doses of a tablespoonful three times daily, and this medication was continued until February 28th, when he was discharged with a well healed wound and improvement of anemia. The reports of the blood examinations were as follows:

February 5, 1903, hemoglobin 65 per cent., reds 2,362,000, whites 5,900. February 28, 1903, hemoglobin 70 per cent., reds 3,800,000, whites 7,000.

CASE 10. L. M., born in the United States, aged twenty-five years, was admitted to the hospital January 3, 1903. She had been married four years, had had one child and one miscarriage. No venereal history. One month before admission she was exposed to cold during menstruation, and the flow ceased. One week before admission she began to flow steadily and still continued to do so, at her entrance to the hospital. She has had severe pelvic pains for three weeks. The uterus was found retroflexed, and a large doughy mass was found on the left side posteriorly. On January 9, 1903, she was operated upon by posterior vaginal section. A suppurating hematocele originating from a ruptured extrauterine pregnancy was found in the left broad ligament. She was given pepto-mangan in doses of a tablespoonful, three times daily, from January 10, 1903, to February 9, 1903. The patient was discharged cured on February 9th. The reports of the blood examinations were as follows:

January 24th, hemoglobin 65 per cent., reds 3,150,000, whites 9,200. February 9th, hemoglobin 75 per cent., reds 4,818,000, whites 6,100.

CASE 11. Mrs. L. G., Italian, twenty-three years of age, married six years, III para, last child three years ago. Admitted January 15, 1903, on the recommendation of her family physician, who had made the diagnosis of ovarian cyst. On admission a careful examination was made and she was found to be pregnant in the eighth month. The woman was delivered in the hospital on February 12, 1903, the labor being normal, but accompanied with considerable hemorrhage, leaving the patient markedly anemic, as she had been previously suffering from anemia during her pregnancy. Pepto-mangan was given her in doses of a tablespoonful three times daily from January 25th to February 28th, when she was discharged cured. The reports of the blood examinations were as follows:

January 29th, hemoglobin 55 per cent., reds 3,126,000, whites 8,450. February 28th, hemoglobin 75 per cent., reds 4,390,000, whites 6,000.

CASE 12. G. G., Italian, forty-four years, single, was admitted to the hospital on November 26, 1902. He is accustomed to smoke a pipe. For the past fourteen months he has had a sore on his lower lip, which gradually grew larger. At times it gave rise to a great deal of pain. On examination, a small growth was found in the median line of the lower lip, hard in consistence, ulcerating, and with a slight infiltration of the surrounding tissues. The sublingual and cervical glands were not enlarged. The growth was removed by a V-shaped incision on December 10, 1902. A moderate degree of anemia remained after the operation, and on February 6, 1903, the patient was given pepto-mangan, in doses of a tablespoonful three times daily. This medication was continued until March 5, 1903, when the patient was discharged cured. The microscopical examination of the growth showed it to be an epithelioma. The reports of the blood examinations were as follows:

February 6, 1903, hemoglobin 70 per cent., reds 3,219,000, whites 8,318. March 5, 1903, hemoglobin 85 per cent., reds 4,890,000, whites 7,000.

On reviewing the results obtained, we find that, considering the diversity of cases studied under the influence of pepto-mangan, the ratio of increase in the hemoglobin and red cells was very uniform. In one case only, 8, of the twelve studied in detail, there was no improvement noted in the anemia, and that was a hopeless case of tuberculous peritonitis, in which, however, the patient was discharged improved as regards her abdominal symptoms after operation. In another case, 6, the improvement was but slight, but this was a patient with renal tumor, and a marked cachexia. These two cases were as severe tests as an iron preparation could be subjected to, and perhaps the paucity of the results is not to be wondered at in these instances.

In the remaining ten cases reported here, as the table shows, the results were very satisfactory for the short duration of the treatment. There is no question that a few weeks longer would have brought most of the "improved" cases up to the point where we could say that the anemia was "cured." But unfortunately our patients belonged to a class in which every day spent in a hospital counts in privations for others who depend upon them, and we have been often obliged, upon the insistent demands of the patients and their friends, to discharge the convalescents at the earliest possible date.

In addition to the forty-odd cases which we studied this winter, pepto-mangan has been used in the hospital for over two years in anemic convalescents, with uniformly satisfactory results. In none of the cases under our observation did any untoward symptom accompany or follow the use of this preparation. In no case did constipation, nausea, headache, or digestive difficulties follow its administration.

The results recorded here correspond with those obtained with the use of pepto-mangan by Loomis[5], Van Schaick[6], and von Ramdohr[7], of New York; Peterson, Perekhan, Doehring[8], of Chicago; Wolffe[9], of Philadelphia; Summa[10] and Bauduy[11], of St. Louis; Von Ruck[12], of Asheville, N. C.; McGuire[13], of Richmond, Va.; Frieser[14] and Pohl[15], of Vienna, and Fasano[16], of Naples.

On the whole, therefore, we have found pepto-mangan a very satisfactory and efficient hematinic in secondary anemias.

REFERENCES.

1. Hammarsten, *Physiological Chemistry*, English Translation, Mandel, New York and London, 1901, p. 176.

2. Bunge, *Zeitschrift f. Physiologische Chemie*, Vol. ix.

3. Quincke, quoted by Häusermann, *Zeitschrift f. Physiologische Chemie*, Vol. xxii.

4. White, *Materia Medica, Pharmacy, Pharmacology and Therapeutics*, 3d Amer. Ed., Edited by Wilcox, Philadelphia, 1895, p. 182.

5. Loomis, H. P., The Successful Treatment of Anemia, *N. Y. Acad. of Medicine, Section on General Medicine*, April 18, 1893.

6. Van Schaick, The Diseases of the Blood in Their Relations to Surgery, and Their Treatment. *New York Medical Journal*, June 2, 1900.

7. Von Ramdohr. Results from Administration of Iron in Readily Assimilable Form after Gynecological Operations. *N. Y. Medical Journal*, June 26, 1897.

8. Peterson, Perekhan, and Doehring. Experimental Reports with Gude's Pepto-Mangan. *Chicago Clinical Recorder*, 1896.

9. Wolffe, A Clinical Report on Gude's Pepto-Mangan, Philadelphia.

10. Summa, The Value of Gude's Pepto-Mangan in the Treatment of Anemia. *New York Medical Journal*, 1895.

11. Bauduy, Observations upon the Treatment of Some Cases of Neurasthenia. *Medical Review of St. Louis, Mo.* February 26, 1898.

12. Von Ruck, K., The Use of Pepto-Mangan for Anemia in Pulmonary Tuberculosis. *New York Medical Journal*, December 15, 1894.

13. McGuire, Surgical Convalescence, with Report of Blood Count in Twenty Cases. *Virginia Medical Semi-Monthly*, 1899.

14. Frieser, Notes on Chalybeate Therapy. The Therapeutic Value of Pepto-Mangan, Guide, Vienna, 1899.

15. Pohl, A Contribution to the Therapeutics of Pepto-Mangan, Gude. *Aerztlicher Central-Anzeiger, Vienna*, September 20, 1899.

16. Fasano, Malarial Anemia, Chlorosis and Rachitis. *Archivo Internazionale di Medicina e Chirurgia*, Naples, May 1, 1899.

---

### LOCAL ANESTHETICS IN AURAL WORK.

In a compilation on local anesthetics in operations on the ear, Dr. Hugo Frey, Vienna (*Internationales Centralblatt fuer Ohrenheilkunde*), claims they were used soon after the introduction into general surgery, but the results have not been as gratifying as in rhinology, laryngology and ophthalmology. He also speaks of the vain attempts to discover an efficient substitute for cocain. In operations on the membrane Kuchner recommends a 10-20 per cent. sol. of cocainum hydrochloricum applied at intervals of five to ten minutes, causing partial anesthesia after fifteen minutes. For paracentesis in acute catarrh, Hedinger uses a 15 per cent., in acute otitis a 20 per cent. solution, but even this causes only a partial anesthesia. Hume and Yeasley report good results with 5-8 per cent. eucain solution, doing away with the unpleasant effects of cocain. Bonain recommends the following: Acidi carbolici crystallisata 2.0, cocain hydrochlorici, menthol, aa 0.5; carbolic acid causing a blanching of membrane, after which paracentesis will be painless, but the large amount of carbolic acid speaks against frequent use of this formula. Gray uses the following: ℞ Cocain muriate 5.0, olei anilini, spiritus vini diluti, aa 15.0, causing complete anesthesia in three to five minutes and disappearance of reflex cough. In order to overcome bad effects of anilin, Gray proposes having a 20 per cent. alcoholic cocain solution and 15 to 20 per cent. eucain solution in anilin oil, mixing equal quantities of both and applying same to membrane with cotton tampon; but even with this formula a pale violet discoloration of membrane takes place. Ethyl chloride spray to the membrane, according to Bloch, is not to be recommended, the application to membrane being painful and necrosis sometimes following the freezing. McAuliffe proposes the injection of cocain solution through eustachian catheter, the nerves of membrana tympani being situated in middle and inner layer, but catheterization is not to be recommended in acute otitis.

In operations of tympanic cavity, as removal of polyps, Frey recommends injections of 1 to 3 per cent. cocain solution into polyps, causing anesthesia in from two to three minutes. The best results are obtained from the use of adrenalin chloride combined with cocain, making the operation a bloodless one. Moure and Brindel use a solution of adrenalin 1:5000 (10.0), cocain 1:10 (5.0). In operations on the auricle, Schleich's method of local anesthesia gives uniformly satisfactory results. In operations on the mastoid where general anesthesia is not advisable ethyl chloride spray and Schleich's infiltration method, preceded by an injection of morphine, are highly recommended. All authors claim that the most painful part of the operation is the incision of the soft parts and detaching the periosteum. Of the methods recommended for local anesthesia Schleich's has taken foremost rank. Braun recommends the substitution of eucain B for cocain in Schleich's formula and uses the following: Eucain B 1.0, saline solution 8.0, aqua 1000; solution must be of body temperature. Eucain is less poisonous and irritating than cocain. Alexander reports eleven cases in which the mastoid was opened under administration of Schleich's local anesthesia. In six of the cases slight headache and vomiting followed the operation, but it is doubtful whether these symptoms were due to Schleich's solution or the result of the concussion. The operation is considerably prolonged by the use of local anesthesia. Whenever possible general anesthesia should be used.—*Medical Fortnightly.*

---

### THE KNOCK-OUT BLOW IN ATHLETICS.

The increasing interest in athletic sports furnishes an argument in favor of instruction in anatomy and physiology in the public schools. A knowledge of certain organs, their function, the effect of fatigue and the position of the body when a blow is received would be information valuable to any one, but especially to those who engage in athletic sports. During exhaustion a blow may produce death, and yet under other circumstances be perfectly harmless, except so far as it may cause pain, or a moment of unconsciousness. The knock-out blow of the prize ring occurs frequently but only occasionally is it fatal.

Doubtless every prize-fighter in the country has at some time been instantly collapsed but most of them recover easily, and the fact that many of them suffer from ill health is attributable to other causes. The reason for such results seldom occur to the casual reader of the daily press.

The June number of the *International Journal of Surgery* quotes from an article in the *British Medical Journal* in which Dr. Duncanson, who has made a study of the various parts of the body concerned in knock-out blows, says that exhaustion is a complicating element. The blows enumerated are on the point of the jaw, producing cerebral concussion; on the temple, laceration of brain substance; angle of jaw, concussion and fracture at base of brain; ear, rupture of drum; kidney blows may be painful but if exhaustion is not present may not be serious.

When the head is thrown back, blows on the neck over the carotid region or over the larynx or hyoid bone may have dangerous consequences, due to the concussion of important nerve trunks. Blows over the region of the heart, especially if delivered while the muscles are relaxed, have produced syncope and even death. The blow on the " mark," or so-called solar plexus blow, is probably the most distressing and dangerous of all. It is inflicted in a region forming a triangle whose apex is at the xypho-sternal articulation, and whose base is formed by a line running between the bony ends of the seventh ribs below. Its disastrous consequences are not due primarily to the effect of the blow upon the solar plexus itself, but to disturbance caused by violent irritation of the stomach, producing shock not only to the solar plexus but also to the vagi. The treatment of this class of injuries is warmth, stimulation and quiet, as in other forms of shock, while special surgical procedures may be indicated in certain cases with cerebral or visceral traumas.

The study of the effects of such injuries is of value, since the accidents both of sport and of industrial life often reproduce conditions similar to those investigated by Dr. Duncanson.—*Medical and Surgical Monitor.*

---

### DISLOCATION OF THE HIP, CONGENITAL.

All cases should be checked by radiography, and no cure should be spoken of that is not a true anatomical cure. All cases of congenital dislocation under fourteen years of age should be submitted to treatment. In the great majority improvement will result, while in some a true anatomical cure is brought about. In all these cases Lorenz's manipulations should be tried in the first instance. Even should they fail to effect reduction, they facilitate any subsequent procedures. The prospect of a cure by Lorenz's "bloodless method" is in direct proportion to the youth of a child, and after the age of four years there is little hope of a true cure by its means. In any case, the chances of a true cure by the "bloodless method" are not very great.

An open operation should be done whenever a radiograph shows that the bloodless method has failed to reduce the dislocation, except, perhaps, in the case of a very young child—under three years—in whom the manipulations may be repeated. With the open operation no fear need be entertained of shock, bleeding or sepsis. Under no circumstances should the joint surfaces be remodeled. An open operation is more calculated to result in a cure, as it enables the surgeon to ascertain beyond a doubt when the head of the bone is really in the acetabulum. The open operation is especially suited for cases over four years of age and for those of bilateral dislocation.

After the operation the limb should be put up in the position of maximum stability; in the majority of cases this will be similar to Lorenz's position. The limb should be put up in plaster of Paris immediately, and the casing should take in the flexed knee; this is essential in order to insure stability of the head of the bone. All tense structures should be stretched or tenotomized as a preliminary measure a week or so previous to the open operation. The after-treatment is practically identical with that of the Lorenz method. F. F. Burghard (*British Medical Journal,* August 29, 1903).

---

### HEMORRHOIDS.

By GEO. A. HEWITT, M.D.

The power of Glyco-Thymoline to relieve turgescence is admirably shown in hemorrhoids, especially of the internal variety.

This affection very frequently occasions severe distress; pain, itching and hemorrhage combined render the patient miserable. In some instances they become seriously debilitated. In the earlier periods of the malady a practical cure may be obtained by the use of Glyco-Thymoline as an injection and compress. The preparation is diluted with an equal quantity of water, and of the mixture, from two drachms to one-half ounce are thrown into the rectum by means of a small syringe. This operation is performed two or three times a day. In the intervals it is a good plan to insert a wad of absorbent cotton, saturated with the fluid, into the rectum in order to secure a more constant action. The remedy has also been given with success by the mouth in such cases, the dose being a teaspoonful, well diluted.

Cases in illustration of this mode of treatment are:

CASE 1. A man, fifty years of age, of robust build, who had always enjoyed good health and muscular vigor, was seriously afflicted. The tumors were not large but they gave rise to intense, lancinating pain in the rectum, shooting likewise into the urethra. There were frequent hemorrhages. For a month or more the man had been unable to sleep much, and had lost considerable flesh. The use of the remedy as indicated above was followed by a very happy result. The symptoms were rapidly ameliorated, and by the end of the month had entirely disappeared.

CASE 2. In the similar, though less severe, case of a man fifty-two years of age, there was pain and occasional bleeding, but the most prominent symptom was distressing pruritus. All manifestations were fully relieved by the use of Glyco-Thymoline.—*The Medical Bulletin*, March, 1900.

---

### EXOPHTHALMIC GOITER.

As surgical treatment is recognized as the most satisfactory in exophthalmic goiter, so is complete bilateral cervical sympatheticectomy to be considered the operation of choice. The operation should not be performed during the height of psychical irritation or tachycardia, nor by an operator who has not an absolute knowledge of the anatomy of the neck and a large experience in dealing with difficult operative procedures or the means at hand to cope with any emergency. The results of the operation are far better than the other procedures, the mortality is much lower, and, in cured cases, the improvement is permanent. In chronic glaucoma, especially after the failure of iridectomy and sclerotomy, this operation may restore vision completely unless the disease is too far advanced, with absence of light-perception. In recurring attacks of epilepsy sympatheticectomy should be resorted to. The results warrant the operation. Jonnesco has reported twelve cures and four improvements in forty-nine epileptics operated on before 1899. J. B. Deaver (*Annals of Surgery*, August, 1903).

[See the *Monthly Cyclopœdia* for August, page 281, for an outline of the indications for this operation in exophthalmic goiter.—S.]

---

### TREATING CARBUNCLES WITHOUT INCISION.

Dr. Salvatore Gucciardella (in the *Gazetta degli ospedali e delle cliniche* of October 5, 1902) tells of spraying a hot 2 per cent. carbolic-acid solution over the carbuncle, followed by hot antiseptic compresses. The vapor consists of steam from a boiling solution, guided to the part by four long tubes narrower at the end nearest the patient, which is about ten to twenty inches distant from the carbuncle. Screens prevent the dissipation, diverting or premature cooling of the steam vapor before it reaches the patient. Steamings last from one-half hour to an hour, and are repeated three or four times daily, or oftener. The hot compresses are soaked in a 1,000 to 2,000 bichloride solution, or 2 per cent. carbolic acid. Rapidity of cure is in proportion to the frequency and duration of the applications.—*Medical Council.*

---

### Another Sanitarium for Lung Troubles for U. S. Army Officers and Men.

The *Colorado Medical Journal,* September, 1903, says: There is good prospect that the big Montezuma Hotel at Las Vegas Hot Springs (about to be closed by the Santa Fé, because of lack of patronage) will be turned over to the U. S. Government and be used as an Army Sanitarium, especially for officers and soldiers afflicted with pulmonary complaints. The Surgeon-General of the army [who has shown commendable foresight and interest in the better care of the army] favors the establishment of another sanitarium in the Southwest, and believes that the big hotel, with a few improvements, would be the place.

---

### Abortion of Felon by Alcohol under Air Exclusion.

Dr. J. R. Eastman, of Indianapolis, Indiana, claims that a commencing felon will always be aborted by the local application of alcohol under perfect air exclusion. Cotton is saturated with the alcohol and placed about the affected part and a thin rubber fingerstall applied over all. Seventy-two hours usually suffices to give relief and even effect a cure. He learned this in Von Bergmann's polyclinic in 1897, since which time he has not had occasion to lance a single felon, the treatment of which was begun in time by this method.—*Medical Council.*

---

### Fractures, Modern Treatment Of.

The writer concludes that our aim in fractures should be: first, restoration of function; second, maintenance of the symmetry of the member; and these should be accomplished by procedures that are as comfortable to the patient and as little annoying to the surgeon as is consistent with the object desired. H. S. McConnell (*New York Medical Journal and Philadelphia Medical Journal,* October 3, 1903).

